# Impact of chemotherapy-induced amenorrhea in breast cancer patients: the evaluation of ovarian function by menstrual history and hormonal levels

**DOI:** 10.1186/1477-7819-11-101

**Published:** 2013-05-20

**Authors:** Kexin Meng, Wei Tian, Meiqi Zhou, Hailong Chen, Yongchuan Deng

**Affiliations:** 1 Department of Surgical Oncology, The Second Affiliated Hospital, College of Medicine, Zhejiang University, 88 Jiefang Road, Hangzhou, Zhejiang Province 310009, China; 2 Department of Thyroid Breast Surgery, Zhejiang Provincial People’s Hospital, 158 Shangtang Road, Hangzhou, Zhejiang Province 310014, China

**Keywords:** Breast cancer, Chemotherapy-induced amenorrhea, Estradiol, Follicle-stimulating hormone

## Abstract

**Background:**

Chemotherapy-induced amenorrhea (CIA) is one of the most frequent therapy-related adverse events observed in breast cancer patients who have undergone chemotherapy. Although the characteristics of CIA have been studied in Western countries, little is known about CIA in Asian. We conducted a retrospective analysis to assess the characteristics and influencing factors of CIA and its association with menopause in Chinese women who underwent adjuvant chemotherapy for early-stage breast cancer.

**Methods:**

Seventy-three premenopausal women who underwent adjuvant chemotherapy for early stage (stages I to III) breast cancer were analyzed. Patient clinical characteristics, treatment regimes, menstrual information, and serum hormone values were collected retrospectively. Characteristic factors relevant to the onset of CIA and menopause were also estimated.

**Results:**

Approximately 83.6% of patients developed CIA. Older patients (>40 years old) had higher CIA incidence compared with younger patients (*P* <0.0001). The onset of menopause was correlated with age (*P* <0.0001) and tamoxifen use (*P* = 0.0313). On the basis of the Kaplan–Meier analysis, a significant difference was observed in the time of onset of permanent amenorrhea as determined by menstrual history and hormone levels (*P* = 0.0028). In women aged 46 to 49 years, the beginning of permanent amenorrhea was detected earlier via the clinical method than via the hormonal method (2 months versus 23 months, *P* <0.0001). In the analysis of patients ≥50 years old, the median time to detection of permanent amenorrhea was 19 months in the hormonal test and 2 months in the clinical test (*P* = 0.0112).

**Conclusions:**

Age at diagnosis is a predictor of the onset of amenorrhea and transformation into menopause among premenopausal breast cancer patients. Adjuvant tamoxifen therapy substantially affects the onset of menopause. A delay of the onset of serum hormone postmenopausal status was observed compared with clinical symptoms. This interval was approximately 21 months in patients aged 46 to 49 years and 17 months in patients aged over 50 years. This interval is significant in the clinical estimate of the menstrual status.

## Background

Breast cancer accounts for 28% of newly diagnosed malignancies among women [1], and almost 25% of those in Western countries occur in premenopausal females [2]. Conversely, the majority of newly diagnosed breast cancers in China are premenopausal females (with age ranging from 40 to 49 years) [3]. As more breast cancer patients benefit from the widespread use of adjuvant chemotherapy [4], long-term side effects, particularly ovarian suppression, become a major concern among women of reproductive age [5]. Ovarian function assessment after chemotherapy in premenopausal or perimenopausal women with hormone-receptor-positive breast cancers is critical in clinical decision making, particularly in cases involving primary adjuvant endocrine therapy. More than one-half (52.2 to 77.7%) of the number of premenopausal women experience chemotherapy-induced amenorrhea (CIA) [6,7], which results in infertility, psychological distress, and prolonged exposure to risks of menopause.

Patient age, intensity of chemotherapy, and tamoxifen dosage are primary determinants of CIA. Older women (>40 years) have a higher risk of developing amenorrhea after adjuvant chemotherapy [8-10]. Previous reports indicated the relevance between currently used chemotherapy regimens (anthracycline-based, taxane-based, and cyclophosphamide/methotrexate/fluorouracil (CMF) regimens) and the onset of CIA [9,11-14]. The occurrence of amenorrhea is frequently associated with the cumulative dosage of chemotherapy [12,14]. The effect of tamoxifen on menstrual cessation has also been determined in several multi-central randomized controlled clinical trials [13,15,16]. However, most of the evidence comes from women in Western countries. Evidence of the occurrence and characteristic factors of CIA among Asian women remains insufficient.

In this study, we conducted a retrospective analysis to assess the onset of CIA and menstruation resumption. Potential factors relevant to CIA and menstruation resumption were determined by employing univariate analysis. Differences in the evaluation efficacy of ovarian function between two methods (menstrual history and hormonal levels) were also discussed.

## Methods

### Patients and treatments

We conducted a retrospective analysis of newly diagnosed premenopausal breast cancer patients managed at the Second Affiliated Hospital of Zhejiang University between 1 January 2007 and 1 September 2011. Participants were eligible for further analysis if they met the following inclusion criteria: (1) newly diagnosed early breast cancer (stages I to III); (2) premenopausal hormone levels and at least two menstrual periods in the preceding 6 months; (3) periodic follow-up (every 3 months) data, including clinical follow-up of menstrual history and serum hormone levels. Patients with gonadotropin-releasing hormone (GnRH) agonist administration or hysterectomy/bilateral oophorectomy histories were excluded.

All patients received four to six cycles of systemic chemotherapy (including anthracycline-based or taxane-based regimens). Patients with hormone-receptor-positive breast cancers were given tamoxifen as endocrine therapy after the termination of adjuvant chemotherapy.

### Data collection

All patients provided verbal consent for their clinical information to be reviewed by investigators. Clinical data (including patient age, tumor stage and size, nodal stage, estrogen receptor, progesterone receptor and Her-2/neu status, chemotherapy regimen and dosage, radiation therapy, trastuzumab administration, and endocrine therapy) was collected from electronic medical records at the Second Affiliated Hospital of Zhejiang University. Information on menstrual history, relapse, or metastasis status was obtained during each follow-up.

Serum estradiol (E2) and follicle-stimulating hormone (FSH) were tested by using chemiluminescence immunoassay via the ADVIA Centaur System (Siemens Diagnostics, Tarrytown, NY, USA). According to the protocol of the chemiluminescence immunoassay kit, postmenopausal hormone levels are defined as FSH level >21.7 IU/L and E2 <110 pmol/L. Premenopausal levels are defined as FSH ≤21.7 IU/L and E2 ≥110 pmol/L. Data on serum E2 and FSH levels was gathered from electronic medical records. The latest serum E2 and FSH levels were selected to determine the menstrual status of the patients.

CIA was defined as amenorrhea that occurred within 6 months after the first cycle of chemotherapy. Menstrual histories provided by participants were used to determine clinical ovarian function regardless of hormone levels. Permanent amenorrhea was defined as having no history of menstrual bleeding after CIA during follow-up. If both E2 and FSH returned to premenopausal levels after the occurrence of CIA, the condition was defined as “harmonic reversible amenorrhea,” regardless of menstrual bleeding. Similarly, menopause was defined in terms of E2 and FSH levels simultaneously within postmenopausal hormone levels after the occurrence of permanent amenorrhea.

### Statistical analysis

Fisher’s exact tests and chi-square tests were employed to evaluate the correlations between amenorrhea and other variables, including age at diagnosis, surgery treatment, chemotherapy regimen, radiation therapy, adjuvant endocrine therapy, and trastuzumab treatment. The chi-square test was used to assess the difference between the groups of included and excluded patients. The rates of amenorrhea of the specified episode (6, 12, 24, and 36 months after initial chemotherapy) were calculated via Fisher’s exact tests.

Among the patients with CIA, the proportions of certain menstrual patterns (reversible amenorrhea or menopause) between treatment regimens and age groups were also assessed via Fisher’s exact tests and chi-square tests. The Kaplan–Meier method was conducted to compare the differences of amenorrhea duration between age groups. The duration between chemotherapy beginning and menstruation resumption determined by clinical definition or hormone measurement was examined via the Kaplan–Meier method. The time of onset of permanent amenorrhea determined by the aforementioned two methods was also evaluated via the Kaplan–Meier method. All statistical tests were two-sided, and *P* values <0.05 were considered statistically significant.

## Results

### Patient characteristics

We reviewed the medical records of 368 premenopausal breast cancer patients who received radical surgery (including modified radical mastectomy and breast-conserving surgical operation) and systemic chemotherapy at our institution. Among the patients, 295 were excluded from the study for the following reasons: 275 patients had insufficient hormone records, 12 patients received GnRH agonist administration after breast cancer diagnosis, 48 patients received bilateral oophorectomy or hysterectomy, 12 patients lack information on their menstruation status, and 27 patients failed to follow-up (several patients were excluded for two or more of the aforementioned reasons). Consequently, 73 patients were eligible for analysis. The median follow-up duration was 27 months (ranging from 10 months to 52 months).

The median age of the 73 patients was 44 years (the age range was 27 to 55 years). The characteristics of patients are shown in Additional file 1 (Table S1). The majority (67.1%) of the patients were aged between 40 and 49 years old. Most of the patients (45.2%) received chemotherapy, including both anthracycline and taxane. A total of 64 patients (87.7%) received tamoxifen as adjuvant hormone therapy. The most significant patient clinical characteristics (for example, age, chemotherapy regimens and tamoxifen intake) in the included group (73 patients) and in the excluded group (295 patients) were almost the same, as shown in Additional file 1 (Table S1).

### Analysis of change in either menstrual history or serum hormone levels

A total of 61 women (83.6%) developed CIA after initial chemotherapy. Among the patients who experienced amenorrhea, CIA occurred more frequently in those older than 40 years (*P* <0.0001). The incidence of menstrual cessation is statistically correlated with age at diagnosis (*P* <0.0001), whereas the types of chemotherapy regimen, tamoxifen intake, trastuzumab treatment, and radiation therapy were not associated with CIA development (see Additional file 1, Table S2).

**Figure 1:**
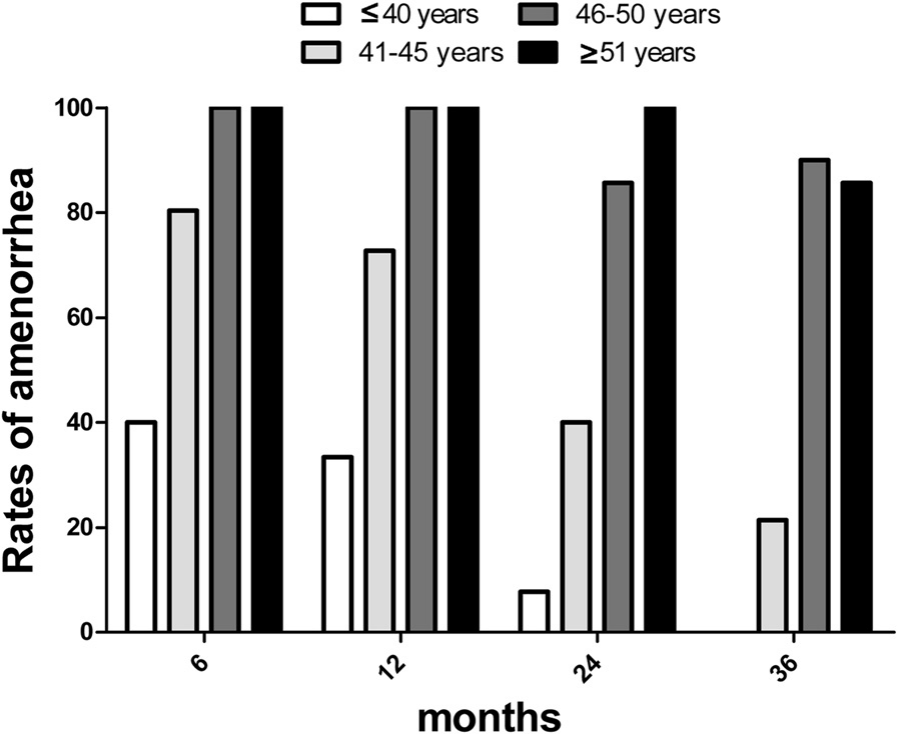
**Age is a critical factor influencing the onset and persistence of chemotherapy-induced amenorrhea.** The rate of amenorrhea in a single age group at a certain follow-up duration is determined based on the following formula: rate of amenorrhea = (number of patients who experienced amenorrhea in a particular age group)/(total number of patients in that age group with available follow-up information).

Among the women who experienced CIA, 28 experienced menstruation resumption in the follow-up periods. The probability of vaginal bleeding was more significant in the younger group (≤45 years) than in others (>45 years) (*P* <0.0001). The median time of menstruation resumption after amenorrhea was 7 months (the range was 3 months to 17 months). The percentage of CIA in young patients (≤45 years) declined after chemotherapy. However, the trend was not remarkable among old patients (>45 years). During each succeeding period, patients >45 years old usually maintain amenorrhea status once CIA occurred (see Figure 1).

Combined with hormone tests, only 22 patients (30.1%) reached postmenopausal serum hormone levels after the occurrence of CIA during the follow-up period. Patients younger than 45 years at diagnosis were less likely to develop menopause compared with those older than 45 years (*P* <0.0001). Patients on tamoxifen therapy had higher risk of menopause regardless of age (*P* = 0.0313) (see Additional file 1, Table S3).

Nine patients exhibited persisting amenorrhea status although their hormone values were under premenopausal levels (see Additional file 1, Table S4). On the basis of the data presented in Additional file 1 Table S4, age at diagnosis statistically affects the incidence of CIA, restoration of menses, and menopause.

### Differences in ovarian function evaluation through menstrual history and serum hormone levels

**Figure 2:**
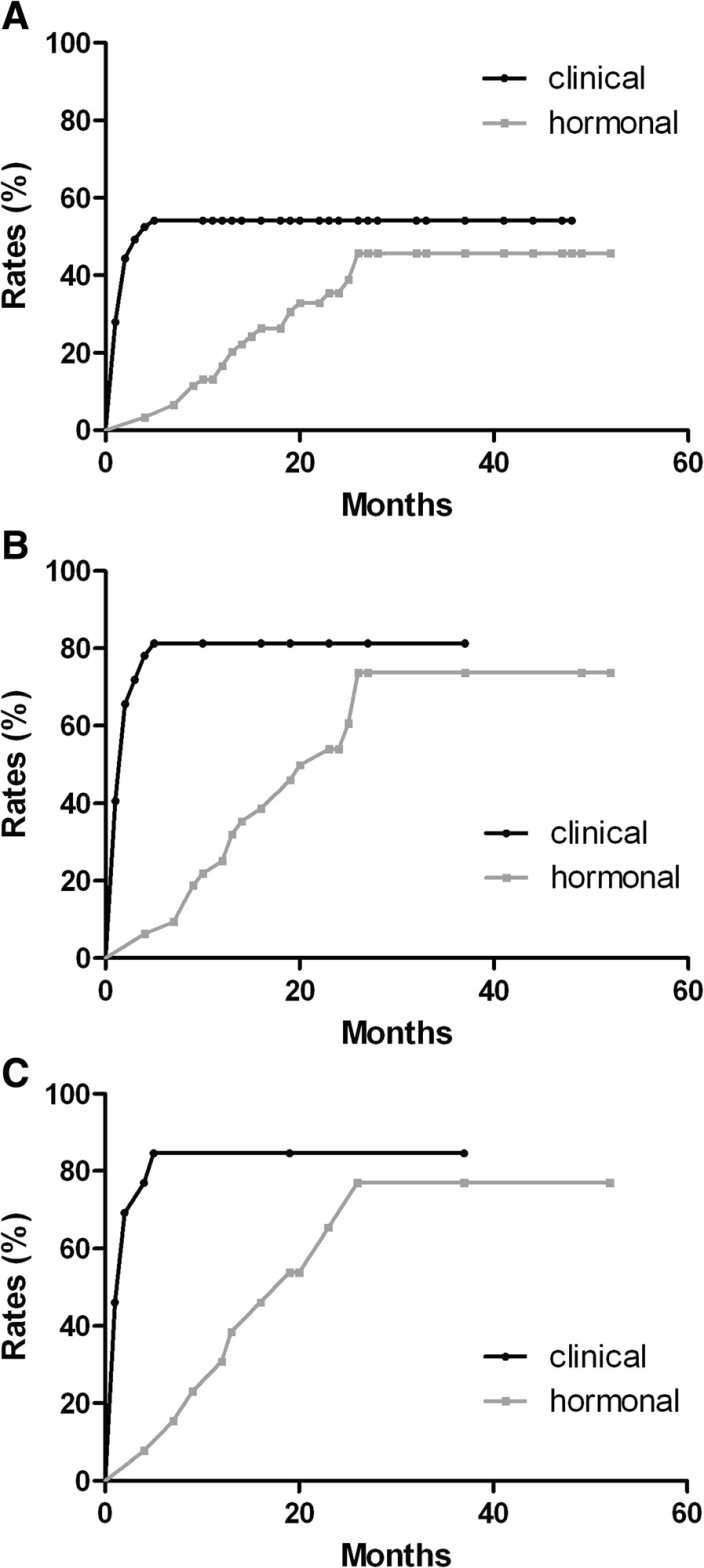
**Clinical cessation of menstrual bleeding is an event occurring earlier than attained serum postmenopausal hormonal levels.** Rates of permanent amenorrhea determined by clinical history and serum hormonal levels in **(A)** general patients, **(B)** patients aged 46 to 49 years, and **(C)** patients aged ≥50 years old.

Among the patients who experienced CIA, many maintained amenorrhea clinically without the simultaneous biochemical evaluations of ovarian depression. Kaplan–Meier analysis results showed a significant difference in the time of onset of permanent amenorrhea, which was determined by menstrual history and hormone levels (*P* = 0.0028) (see Figure 2). In women aged 46 to 49 years, the beginning of permanent amenorrhea was detected earlier according to the clinical method than according to the hormonal method (2 months versus 23 months, *P* <0.0001) (see Figure 2). In the analysis of patients ≥50 years old, the median time to detection permanent amenorrhea was 19 months in the hormonal test, and 2 months in the clinical test (*P* = 0.0112) (see Figure 2).

**Figure 3:**
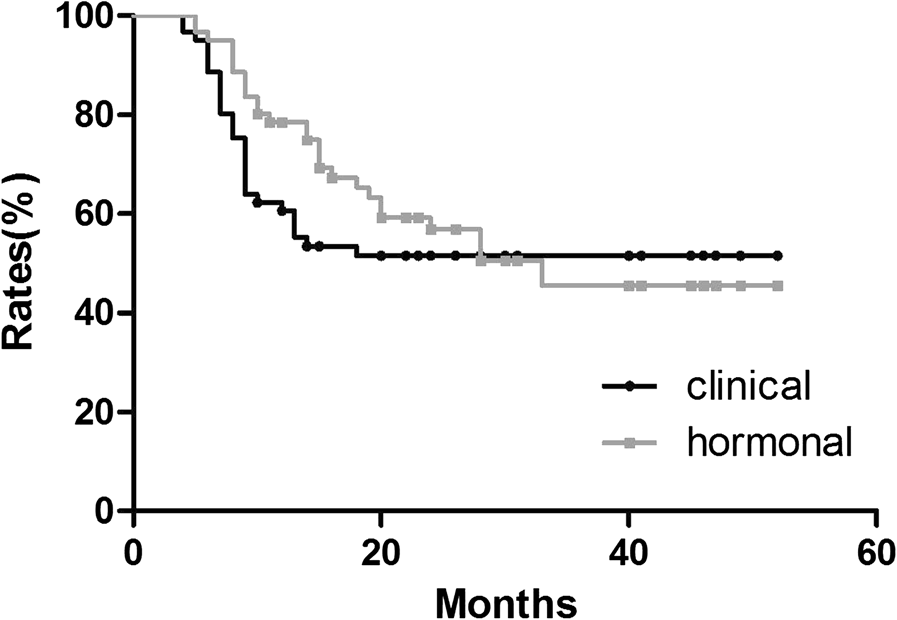
No difference in ovarian depression duration was detected between menstrual history and hormonal method.

**Figure 4:**
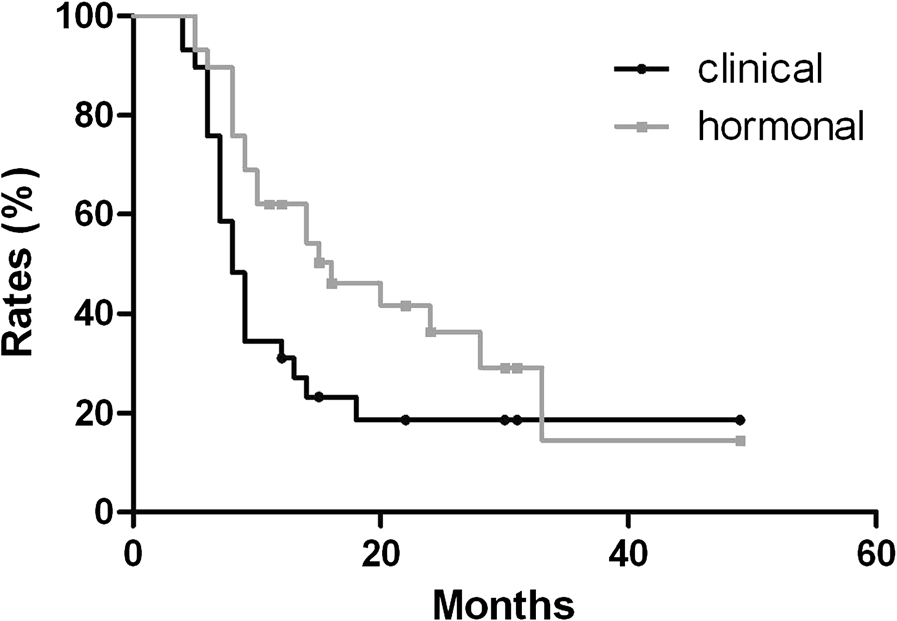
Clinical resumption of menstrual bleeding is an event occurring earlier than attained serum premenopausal hormonal levels in determining ovarian depression after chemotherapy-induced amenorrhea in patients aged ≤45 years.

In patients who regained ovarian function, no difference in ovarian depression duration was detected between menstrual history and the hormonal method via Kaplan–Meier analysis (see Figure 3). However, in the group younger than 45 years old, the median ovarian depression duration was shorter via the clinical method than that via the hormonal method (8 months versus 16 months, respectively, *P* = 0.0101) (see Figure 4). No difference was detected between the two methods among the group of patients older than 45 years.

## Discussion

CIA is one of the frequent therapy-related adverse events observed in patients who undergo chemotherapy. CIA is a significant drain on the quality of life of cancer patients because of its associated menopausal symptoms and potential risk of infertility. In this study, we conducted a retrospective analysis to assess the onset of CIA and menstruation transformation among Chinese women who underwent chemotherapy for breast cancer. A total of 73 premenopausal breast cancer patients were included in this study, whereas 295 patients were excluded for various reasons. The major clinical characteristics in the included group (73 patients) and in the excluded group (295 patients) were almost the same. Although only 19.8% of the potential group is included in our analysis, the potential selection bias is limited.

In this study, the rate of CIA (83.6%) was similar to the data provided by other trials. In the International Breast Cancer Study Group trial 13–93 [17], the general incidence of CIA was 86%, which is approximately equal to our results. Han and colleagues reported that the rate of CIA in the Korean population was 89.1% [13]. However, the incidence of CIA was lower (68%) in the findings of Lee and colleagues [16]. The differences may have resulted from the different age compositions among these studies. The higher component ratio of included patients who were ≥40 years old (78.1% in our study versus 61% in the study of Lee and colleagues) may be attributed to the higher rate of CIA onset in our study.

Previous evidence indicates the incidence of CIA depends on the patient age or type and intensity of chemotherapy or tamoxifen dosage [18]. In this study, we also discussed these potential factors on the onset of CIA and the transformation to menopause via univariate analysis. Our study demonstrated that age at diagnosis is an important influencing factor on the onset of CIA, which is in accordance with previous studies [7,17,19-22]. Age at diagnosis was also a major predictor for both reversible amenorrhea and menopause, as shown in Additional file 1 Table S4. Additionally, we found that Chinese women younger than 40 years who experienced CIA had higher incidence of menstruation resumption. Similar results were reported in other Asian-based studies [13,16], as well as in Western studies [6,22]. Patients aged ≤45 years old were less likely to develop menopause after CIA and more likely to experience menstruation resumption or maintain amenorrhea, as confirmed by both clinical manifestation and serum hormone status in the study.

Previous evidence indicates that tamoxifen is a potential influencing factor of CIA onset. In this study, the relevance between the administration of tamoxifen and CIA was not significant in the univariate analysis. However, this relevance was influenced by the age of the patient at diagnosis [20,22]. Given the limited number of patients in different age-based subgroups, conducting subgroup analysis, as well as determining the association between the tamoxifen and patient age, is difficult. Our data indicated that the addition of tamoxifen substantially affected the onset of menopause. The possible explanations are the disturbance of negative feedback from the hypothalamus to the ovary [18] and the depression of pituitary production triggered by tamoxifen [23,24].

Menstrual status determined by the history of menstrual bleeding is a conventional and convenient method of assessment in determining ovarian function [9]. However, menstrual status cannot thoroughly reflect actual ovarian function [25]. The serial measurement of serum ovarian biochemical markers is an acceptable method to reflect ovarian function. Accordingly, in this study, we determined patient menstrual status by employing both clinical menstrual history and serum ovarian biochemical markers. We compared the differences in the median time to onset of permanent amenorrhea between the aforementioned two approaches. Our results indicate that the time of clinical cessation of menses was earlier than the recorded serum postmenopausal hormonal levels. As shown in our study, for Asian patients aged between 46 and 49 years, another 21 months were needed for hormone levels to reach the postmenopausal range after the cessation of menses. For patients older than 50 years, another 17 months were necessary. The World Health Organization defined 12 months of amenorrhea as an indication of menopause [26]. On the basis of our results, this criterion is not reasonable for identifying women who experienced premature amenorrhea after cytotoxic therapy. Accordingly, the serial measurement of serum FSH and E2 combined with menstrual history are suggested as an assessment for ovarian function [27]. Given the complexity of ovarian change after chemotherapy, the duration of hormone measurement after amenorrhea onset for postmenopausal status confirmation is uncertain. The guidelines provided by the National Comprehensive Cancer Network suggest a postmenopausal range of 12 months to meet the criteria for menopause. However, most studies support the regular monitoring of hormone status without mentioning the duration [27,28]. We propose that the clinical history and serum hormone level follow-up duration should last for at least 21 months for patients aged 45 years to 49 years and 17 months for patients aged ≥50 years to determine the exact menstrual status among Asian women.

Previous studies preferred FSH as a biochemical marker to predict ovarian reserve [26]. The role of FSH in accessing ovarian function has been well demonstrated. Other than FSH, anti-Müllerian hormone (AMH) and inhibin B are also shown to improve the predictive capacity of ovarian function [29,30]. AMH and inhibin B appear to be superior to FSH because they are not affected by the administration of tamoxifen [30]. Moreover, AMH and inhibin B reflect subtle changes in menstrual transition, which has been compared with FSH. AMH is also strongly recommended for its sensitivity in predicting ovarian function and stable expression over the menstrual cycle [25]. With regard to biophysical parameters, antral follicle count (AFC) is also a useful parameter. The function of AFC in assessing ovarian function among children cancer survivors has been proven [31]. Reduced AFC was observed in breast cancer patients after chemotherapy and appeared to be in accordance with suppressed ovarian function [25]. Further investigation is necessary to confirm the function of such biomarkers in clinical application.

Our study had several limitations, such as a short follow-up period and small sample size. As previously stated, the large proportion of patients dropping out of the study was assumed to have resulted from the lack of periodic hormone values. Approximately 26% hormone insensitivity and poor adherence would be the possible reasons for the missing information, which introduced bias. The small number for certain groups (for example, patients without amenorrhea) can affect the precision of CIA rates in perimenopausal breast cancers. As a retrospective study, the menstrual status might be less reliable due to a recall bias. Despite these limitations, our study is an effective exploration of time points and intervals between them to predict the ovarian function pattern during follow-up. A more comprehensive study with a longer follow-up period and written diaries of patients is planned by our institution in this area. In addition, other hormone markers, including AMH and inhibin B, could be assessed serially in determining post-chemotherapy ovarian reserve, and the related cutoff point for these biomarkers could be evaluated.

## Conclusions

An accurate and probable evaluation of ovarian function promotes better understanding of the effect of cytotoxic agents, thereby offering cancer patients useful information with respect to appropriate endocrine therapy and fertility preservation.

## Abbreviations

AMH: Anti-Müllerian hormone; AFC: Antral follicle count; CIA: Chemotherapy-induced amenorrhea; E2: Estradiol; FSH: Follicle-stimulating hormone; GnRH: Gonadotropin-releasing hormone.

## Competing interests

The authors declare that they have no competing interests.

## Authors’ contributions

KM carried out the data collection and drafted the manuscript. WT participated in the design of the study. MZ carried out the follow-up work. HC conducted the statistical analysis. YD conceptualized the study, participated in its design and coordination, and assisted in drafting the manuscript. All authors read and approved the final manuscript.

## Supplementary Material

Additional file 1 **The file name Table-wjso.xls includes the following tables: Table 1.** Patient characteristics; **Table 2.** Univariate analysis of CIA; **Table 3.** Univariate analysis of menopause; **Table 4.** Age group and menstrual status. This file refers to a multi-page table for the paper. All tables are shown in Sheet 1. Click here for file
